# Can social factors influence the dietary restraint of girls as young as five?

**DOI:** 10.1186/2050-2974-3-S1-O23

**Published:** 2015-11-23

**Authors:** Stephanie R Damiano, Susan J Paxton, Eleanor H Wertheim, Siân A McLean, Karen J Gregg

**Affiliations:** 1 School of Psychology & Public Health, La Trobe University, Australia

## 

Dieting is a risk factor for the development of eating disorders, and research suggests that dietary restraint emerges from a young age. There is a need to understand factors associated with the development of dieting intentions to prevent later disordered eating. The aim of this research was to explore individual and sociocultural factors related to 5-year-old girls' dietary restraint. Participants were 111 5-year-old girls, interviewed about their dietary restraint, body image, appearance ideals, positive weight bias, and peer conversations. Their mothers (N = 109) completed self-report questionnaires assessing dietary restraint and appearance ideals, and measures assessing their daughter's media exposure and peers' appearance interest. A moderate level of dietary restraint was reported by 34% of girls, half showed internalisation of the thin ideal, while the majority were satisfied with their body size. Higher levels of girls' dietary restraint were associated with attributing positive characteristics to thinner figures, greater internalisation of the thin ideal, more media exposure, and greater appearance conversations with peers. Sociocultural factors, including media exposure and peer conversations, were stronger predictors of dietary restraint than individual factors, suggesting that girls may have a tendency to diet due to social pressures rather than dissatisfaction with their size.

